# Book Review: Conducting a Culturally Informed Neuropsychological Evaluation

**DOI:** 10.3389/fpsyg.2017.00993

**Published:** 2017-06-15

**Authors:** Yannick Gounden

**Affiliations:** CRP-CPO, EA7273, Université de Picardie Jules VerneAmiens, France

**Keywords:** cross cultural, neuropsychology, assessment, ethics, individual differences

In a context of globalization where society is increasingly multicultural and where scientific collaborations are highly encouraged at an international level, taking multiculturalism into account is essential for both psychologists and researchers in psychology. The work of Daryl Fujii on how to conduct a culturally informed neuropsychological evaluation is a must read book, not only for neuropsychologists but also for anyone who has an interest for brain-behavior relationships in culturally different populations (an increasing trend in many developed countries). There is indeed a growing literature on cross-cultural neuropsychology but to my knowledge, the present book is the first comprehensive integrated resource on the challenges of cross-cultural diversity in the field of clinical neuropsychology. It complies both conceptual and practical elements with up to date references and gives informative examples in echo with the experiences of any neuropsychologist (clinician or researcher).

The book is divided into three parts each composed of 2 to3 chapters (10 chapters in all). In part 1 (Foundations: attitudes, perspective, and conceptual overview) the author insists on the importance of adopting an ethnorelative attitudes “acceptance of the other as different but equal” and warns on the impact of more ethnocentric attitudes “considering the other within one's own cultural lenses and values with the assumption of superiority.” To illustrate how the attitudes of clinicians (i.e., the degree his/her intercultural sensitivity) can affect neuropsychological assessments, the author introduces Bennett's (1993) developmental model of intercultural sensitivity. This model interestingly illustrates how along the continuum of six stages (from the highest level of ethnocentricism to ethnorelativism), assessments of patients can be biased in various ways. I believe that this idea of continuum more easily allows psychologists or and even researchers, to consider their own practice, something that would have been more complicated in a more Manichaean approach. Another highlight in the first part of the book is the reference to various sources of guidelines usually followed by psychologists and researchers such as the American Psychological Association (2010). By providing a synthesized version of the APA Ethics Code (American Psychological Association, 2011) and multicultural Guidelines for Assessments (American Psychological Association, 2003), the author presents general strategies for competent assessments. Overall, chapters 1 and 2 of part 1, introduce the cornerstones on how to conduct a culturally informed neuropsychological evaluation.

Having established the foundations on cross cultural neuropsychology, in the second part of the book entitled “Preparation” the author gives an overview of the various preparations needed prior to conducting a neuropsychological evaluation with a person of different culture. Three preparatory tasks are described in chapter 3 and concern the following topics: contextualizing behaviors and presentations, maximizing cooperation during testing, and facilitating communication. I was personally surprised to come across some factors that can have an impact on the hypothesis development and test selection, but too frequently neglected in clinical practice. Indeed, if we consider “communication” for instance, many psychologists consider the verbal language barrier when interacting with a person of different culture. Generally, the help of an interpreter is requested and results of the assessments are interpreted with caution. However, Daryl Fujii also insists on the importance of gathering information on “non-verbal convention” in a particular culture “What are norms for interpersonal distance, eye contact, and pace of exchange? What are common nonverbal gestures?” A comprehensive review of native Indian and Alaskan native cultures is proposed to illustrate the importance of taking into consideration various cultural factors that can impact on the interaction between the psychologist and the person of a different culture. Although this illustrative case speaks more to clinicians of the United States, the elements that everyone can extract is universal and can apply to other countries. Chapters 4, 5, 6, and 7 give practical recommendations and possible solutions to typical problems encountered by psychologists when conducting neuropsychological assessments with people not belonging to the mainstream culture. Various potential treats to validity are identified and strategies for interpreting test results in a way that meets International test commission recommendations are proposed. The author also addresses a recurrent issue for psychologists and researchers in neuropsychology, “How to estimate premorbid functioning of patients with different cultures?.” No concrete solution exists but the authors proposed in a non-dogmatic way some interesting ways to deal with this headache. Finally, part II of the book ends with psychometric considerations and recommendations for test selection followed with a list of neuropsychological tests that have demonstrated cross-cultural validity along with references for validation studies describing norms from different countries. Many of these tests are unfortunately available only in English language but this inventory can be useful for researchers who would like to adapt in their language, tests that have robust cross-cultural validity.

In a logical flow the last part of the book illustrates what a culturally informed neuropsychological process entails in actual practice. The author presents a comprehensive case sample (Jae Song Kim, a 66-year-old Korean American man who sustained a right–sided basal ganglia stroke). The case of Mr Kim is well-exposed and shows the importance of careful preparation prior to the assessment. The guidelines for writing a report or for conducting a feedback session with a patient and his or her family of different culture are instructive and I found them well-fitted with regard to the emerging collaborative and patient-centered approach in clinical neuropsychology (Gorske and Smith, 2008).

Although this book is written as a step-by-step manual providing a broad framework for performing culturally informed neuropsychological evaluations, this is in fact not the case. Instead, the book intends to bring psychologists and researchers to understand the importance and complexity of conducting a culturally informed neuropsychological assessments. Solutions are not given to solve problems and instead the book can help the psychologist to find his or her own solutions with regard to the singularity of a given situation. The aim to encourage reflection is apparent since each chapter starts with a quote. My favorite one is from chapter 9 by Plato “A good decision is based on knowledge and not on numbers.”

## Author contributions

The author confirms being the sole contributor of this work and approved it for publication.

### Conflict of interest statement

The author declares that the research was conducted in the absence of any commercial or financial relationships that could be construed as a potential conflict of interest.
